# Resignation of Prof. Trousseau

**Published:** 1864-01

**Authors:** 


*Resignation of Professor Trousseau.*—Prof. Trousseau has offered his resignation as Clinical Professor in the Faculty of Medicine which he has so ably filled for upwards of thirty years, being, he says, now turned of 62, and because to perform the duties of the chair, as he has always done, will demand an amount of labor for which his strength and activity will not much longer suffice. The students, however, are not willing to part with their professor, and have petitioned the Minister of Public Instruction not to accept the resignation.

